# Role of microRNAs in Arbovirus/Vector Interactions

**DOI:** 10.3390/v6093514

**Published:** 2014-09-23

**Authors:** Sassan Asgari

**Affiliations:** Australian Infectious Disease Research Centre, School of Biological Sciences, The University of Queensland, Brisbane, QLD 4072, Australia; E-Mail: s.asgari@uq.edu.au; Tel.: +61-7-336-52043

**Keywords:** mosquito, arbovirus, gene regulation, microRNA

## Abstract

The role of microRNAs (miRNAs) as small non-coding RNAs in regulation of gene expression has been recognized. They appear to be involved in regulation of a wide range of cellular pathways that affect several biological processes such as development, the immune system, survival, metabolism and host-pathogen interactions. Arthropod-borne viruses impose great economic and health risks around the world. Recent advances in miRNA biology have shed some light on the role of these small RNAs in vector-virus interactions. In this review, I will reflect on our current knowledge on the role of miRNAs in arbovirus-vector interactions and the potential avenues for their utilization in limiting virus replication and/or transmission.

## 1. Introduction

Arboviruses (arthropod-borne viruses) replicate in arthropod vectors and are transmitted through blood feeding to vertebrate hosts, in which they also replicate. They cause widespread deadly diseases, morbidity and mortality, imposing great threats to human and animal health globally [1]. Arboviruses are mainly RNA viruses and belong to the families *Flaviviridae* (+ssRNA), *Togaviridae* (+ssRNA), *Bunyaviridae* (-ssRNA), *Rhabdoviridae* (-ssRNA) and *Reoviridae* (dsRNA) [2]. Mosquitoes, midges, sandflies or ticks are the main vectors of these viruses. Replication of arboviruses in the arthropod host is relatively harmless to the vector to presumably avoid compromising the vector’s overall fitness and fecundity. This requires tight regulation of virus replication and host genes to prevent overt cytopathologic effects by establishing an optimal balance between virus replication and subversion of the host anti-viral responses.

MicroRNAs (miRNAs) are ~22 nucleotide (nt) non-coding RNAs that play significant roles in various biological processes through regulation of gene expression at transcriptional or post-transcriptional levels. Several studies have shown differential expression/abundance of host miRNAs following viral infection. These are usually determined through microarray analysis or more recently deep sequencing of small RNAs from non-infected and infected samples followed by validations. Differential expression of host miRNAs could be either due to anti-viral responses or regulatory factors produced by infecting viruses. Considering that each miRNA may have several targets and each target might be regulated by more than one miRNA, a composite network of gene regulation could be envisaged making the study and interpretation of miRNA effects rather complicated.

The discovery of virus-encoded miRNAs added a new dimension to virology and host-virus interactions. They were first discovered in infections with Epstein Bar virus, a herpesvirus [3], followed by identification and characterization of many other virus-encoded miRNAs from herpesviruses as well as other DNA viruses. Recently, there have been a number of publications pertinent to characterization of functional miRNAs or miRNA-like small RNAs from RNA viruses, including arboviruses. In this review, I will briefly touch on miRNA biogenesis with an emphasis on the non-canonical pathways used for the production of miRNAs from RNA viruses, examine the effect of arbovirus infection on the host miRNAs, discuss the role of specific host or virus-encoded miRNAs in arbovirus-host interactions, and finally briefly review the effect of *Wolbachia* as an emerging disease control agent on the host miRNA profile that affects arbovirus-vector interaction.

## 2. Biogenesis of miRNAs 

### 2.1. Canonical Pathway

**Figure 1:**
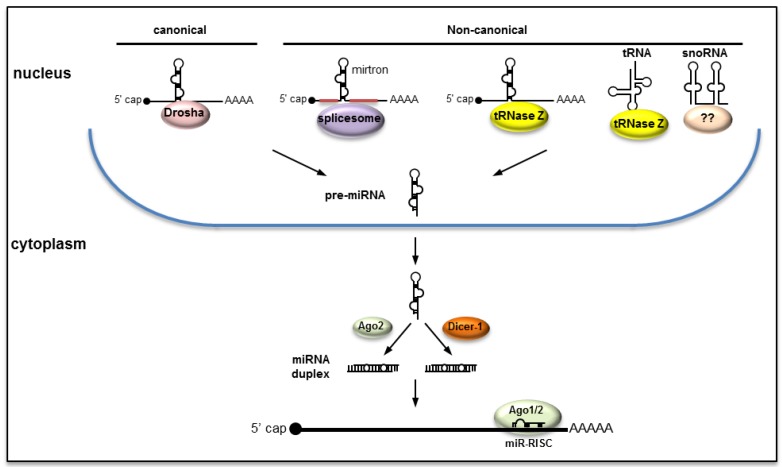
miRNAs are produced through canonical as well as non-canonical pathways.

Cellular miRNAs and those encoded by DNA viruses that replicate in the host nucleus are usually produced through the canonical pathway (Figure 1). Accordingly, the primary miRNA (pri-miRNA) transcript that has all the characteristics of an mRNA (5’ cap and polyA tail) is transcribed by the cellular RNA polymerase II. miRNAs may originate from exclusively miRNA genes with their independent promoters or may derive from introns and rarely from exons. The pri-miRNA transcript, which may consist of one or more stem-loop structures, is processed by the microprocessor with the major players being Drosha, an RNase III type ribonuclease, and a double-stranded RNA binding protein called Pasha [known as DGCR8 (the DiGeorge syndrome critical region 8) in vertebrates] [4,5]. Drosha excises the precursor miRNA (pre-miRNA) by cleaving the base of the stem releasing a ~70 nt stem-loop, which is subsequently transported into the cytoplasm with the aid of the Exportin-5/Ran protein complex [6]. In the cytoplasm, another RNase III type enzyme, Dicer-1, cleaves the hairpin head of the stem-loop releasing a ~22 nt miRNA 5p:3p duplex (also known as miRNA:miRNA*). The duplex is recruited in Ago1 or Ago2 proteins initiating the formation of the miRNA-RNA Induced Silencing Complex (miR-RISC). Often one of the strands (the passenger strand noted miRNA*) is degraded and the mature miRNA (the guide strand) guides the miR-RISC to target sequences, which are mostly mRNAs (in post-transcriptional gene silencing) or sometimes DNA sequences in promoter regions in the nucleus (in transcriptional gene silencing). In certain instances, both the guide and the passenger strands could function as mature miRNAs by recruitment in both Ago1 and Ago2 and interact with target sequences. In insects, Ago1 was thought to be primarily involved in the miRNA pathway and Ago2 in the RNA interference (RNAi) response. Recent evidence indicates that mature miRNAs could be recruited in both Ago1 and Ago2 [7,8,9,10].

### 2.2. Non-Canonical Pathways

As we learn more about miRNAs, it appears that they could also be produced through pathways that deviate from the canonical pathway (Figure 1). For example: (1) pre-miRNAs could be produced from hairpin introns during splicing, independent of Drosha, producing the so-called mirtrons [11]; (2) miRNAs may originate from tRNAs with cleavage mediated by tRNase Z (such as miR-1983) [12]. tRNase Z was also found to excise pre-miRNAs from pri-miRNAs of a murine γ-herpesvirus without Drosha’s involvement [13]; (3) miRNAs may originate from small nucleolar RNAs (snoRNA), which are primarily localized in the nucleolus and are involved in site-specific methylation and pseudouridylation of other RNAs [14]. In this process, the pre-miRNA is derived from snoRNA independent of Drosha but further processed by Dicer in the cytoplasm.

### 2.3. Biogenesis of miRNAs from RNA Viruses

It was originally believed that RNA viruses might not produce miRNAs. Reasons included: replication of most RNA viruses, including arboviruses, occurs in the cytoplasm and therefore there is a lack of access to the nuclear miRNA microprocessor (Drosha/Pasha) for the conversion of pri-miRNA to pre-miRNA, and the possibility of the viral RNA genome to be targeted by virus-encoded miRNAs that could trigger the RNAi response and degradation of the viral genome. Nevertheless, no miRNAs from RNA viruses that replicate in the nucleus have been reported either; although it has been shown that the cellular miR-124 could be successfully produced from a recombinant influenza A virus expressing the pri-miR-124 through the canonical pathway, without affecting virus replication [15]. This suggests that RNA viruses that replicate in the nucleus could potentially encode miRNAs.

Initially, a number of miRNAs were reported from HIV [16], but they have been treated with skepticism due to irreproducibility of their detection. Further insights into the biogenesis of miRNAs have been gained over the years unraveling the possibility of non-canonical biogenesis of miRNAs without the involvement of the nuclear enzyme Drosha. In addition, a number of studies have shown that when the precursor stem-loops of DNA virus-encoded miRNAs or vertebrate miRNAs were inserted into the genome of RNA viruses, they were successfully processed into mature miRNAs. These suggested that production of miRNAs by RNA viruses could also be possible. For example, when the pre-miR-BART2 from Epstein Barr virus was inserted in the 3’UTR of the flavivirus tick-borne encephalitis virus (TBEV) genome it was successfully processed into mature miR-BART2 without affecting virus replication [17]. In another example, the cellular miR-124 was produced from recombinant Sindbis virus (SINV), a positive strand RNA virus, without miRNA-mediated targeting of viral RNAs [18]. A more recent follow-up study demonstrated that Drosha could be relocalized into the cytoplasm following SINV infection mediating cleavage of pri-hsa-miR-122 and -miR-124 expressed from the engineered SINV into pre-miRNAs [19]. These could subsequently be processed into mature miR-122 and miR-124. The relocalization had no effect on the biogenesis of cellular miRNAs. These examples from cytoplasmic RNA viruses imply that pri-miRNA stem loops could be processed in the cytoplasm without requiring a nuclear event.

In another example, a number of miRNAs were discovered from the retrovirus, Bovine leukemia virus (BLV), produced without Drosha’s involvement in generating pre-miRNAs. Instead, the pre-miRNAs were found to be the transcription products of RNA polymerase III [20]. Sequence analysis of pre-miRNAs confirmed that they lacked Drosha cleavage signatures. This example from RNA viruses further proposes that pre-miRNA stem loops could be produced independent of the nuclear microprocessor.

## 3. miRNA-Target Interaction

Interaction of mature miRNA, loaded in the miR-RISC, with target sequences involves base pairing of miRNA sequences with complementary sequences in the target sequences [21]. While in plants this complementarity is 100%, in animals mismatches are very common. Complementarity of the seed region (nucleotides 2–8 from the 5’ end of mature miRNA) has been regarded as an important criterion for miRNA-target interaction, with a number of target detection software packages heavily relying on this criterion. As more evidence emerges, it appears that lack of complementarity in the seed region could be compensated by complementarity in the centre or towards the 3’ end of the miRNA [22,23]. It is now widely known that one miRNA can bind to many targets and regulate them [21]. Furthermore, due to redundancy in binding sites, multiple different miRNAs may also bind to the same target (e.g., [24]). These phenomena add to the complication of investigating the function of individual miRNAs or target genes that are obviously involved in complex regulatory networks. It is worth mentioning that while bioinformatics analyses help the initial identification of potential targets of each miRNA, experimental validations are required to establish the actual interactions between miRNAs and target gene(s).

Initially, it was thought that target sequences exclusively reside in the 3’UTR region of mRNAs, however, more evidence has accumulated suggesting that target sequences could also be in the 5’UTR as well as in the open reading frame (e.g., [22,25,26,27,28,29]). Another common impression is that miRNA-target interaction always leads to negative regulation of the target by degradation of mRNA or repression of translation. Still many investigators, when combining miRNA profiles with transcriptome data, concentrate on targets with transcript levels that negatively correlate with miRNA abundance. Several studies have shown that miRNA-mRNA interaction may also lead to positive regulation of the target (e.g., [30,31,32,33]). This could either be activation of translation by interacting with promoter sequences or at the post-transcriptional level, for example, by increasing the stability of mRNA. A classic example is the liver-specific miR-122, which stimulates translation of Hepatitis C virus (HCV) and also increases the stability of the genomic RNA. This is achieved by recruitment of the Ago2 protein to the 5’UTR of the virus genome via interaction of miR-122 with the target sequences in the region [34]. An example of a cellular miRNA with a known mechanism of target induction is miR-128. The miRNA targets transcripts of nonsense-mediated decay machinery resulting in increases in the transcript levels of genes involved in brain development [30]. 

## 4. Role of miRNAs in Vector-Arbovirus Interactions

Following the acquisition of an arbovirus by an arthropod vector through a blood meal taken from an infected host, the virus typically infects and replicates in the midgut cells as the primary site of infection. This is followed by the release of replicated virions into the hemocoel in which they circulate via hemolymph and eventually reach and infect the salivary glands where they replicate and are subsequently delivered to the next host upon blood feeding. These events occur over the extrinsic incubation period, which is required for biologically transmitted pathogens. Changes in the abundance of cellular miRNAs appear to be a common consequence following virus infection although its extent may vary. During the extrinsic incubation period, the arbovirus has to overcome or evade the vector’s anti-viral responses but also regulate its replication to presumably avoid compromising the host’s health and fitness. MiRNAs have been shown to be involved in cellular responses, counter measures by viruses and also keeping replication in check. Thus far, examples of miRNA involvement in arboviruses-vector interactions are few, which are discussed below.

### 4.1. Effect of Virus Infection on the Host miRNA Profile

To date, miRNAs from a number of arbovirus vectors, mainly mosquitoes, have been reported. These include *Aedes aegypti* [35], *Aedes albopictus* [36,37], *Anopheles gambiae* [38], *Anopheles stephensi* [39], and *Culex quinquefasciatus* [37]. Differential expression of cellular miRNAs following virus infection has been shown in numerous examples in invertebrates and vertebrates, which could either be due to the host response to infection or virus interference with the miRNA biogenesis. The latter may include virus-encoded miRNAs that interfere with host survival, immunity or proliferation.

In one of the earlier studies, it was shown that West Nile virus (New York strain 99, WNV_NY99_) caused changes only in the abundance of a small number of miRNAs in *Cx.quinquefasciatus* female mosquitoes [37]. These included miR-989 with 2.8-fold downregulation and miR-92 which was conversely upregulated (1.5-fold) following virus infection. These were shown by primer extension in persistently infected C7/10 cells with WNV replicons and deep sequencing of* Cx. quinquefasciatus* mosquitoes infected with WNV_NY99_. In addition to those two miRNAs, four other miRNAs, miR-957, miR-970, miR-980 and miR-33 showed alterations in their abundance in WNV_NY99_-infected mosquitoes but not to the same extent. The investigators did not identify the targets of miR-989 and -92, but speculated that they may have a role in WNV-mosquito interaction.

In *Ae. aegypti*, a comprehensive analysis of miRNAs by deep sequencing based on multiple biological replicates was carried out in which the miRNA profiles of mosquitoes at 2, 4 and 9 days following exposure to dengue virus serotype 2 (DENV-2) were compared with those of control mosquitoes of the same age. The results revealed significant differential abundance of a total of 35 miRNAs upon DENV-2 infection; five at 2 days post-infection (dpi), three at 4 dpi and 27 by 9 dpi [40]. Of these, four were upregulated and the rest were downregulated. Interestingly, the differentially abundant miRNAs at each time point were distinct. *In silico* target prediction, using three prediction methods, determined 4076 targets in the 3’UTR and coding sequence regions in the transcriptome of *Ae. aegypti* with 365 of the targets having targets in both regions. The majority of these targets were categorized into biological functions involved in transport, signal transduction, the cytoskeleton/structure and metabolism, all of which are important in DENV infection. However, miRNA-target interactions need to be experimentally validated and their effects on virus replication explored. 

In *Ae. albopictus*, a number of miRNAs were differentially expressed in C6/36 cells following DENV-2 infection [41]. One of these miRNAs, miR-252, exhibited a greater than 3-fold increase following infection. Inhibition of this miRNA by synthetic inhibitors of the miRNA led only to 1.5-fold increase in DENV genomic RNA replication. The authors suggested that the miRNA might function as part of the host’s anti-viral response; although the fold change in virus RNA replication appeared to be small. The investigators found target sequences of miR-252 in the E protein gene from the virus genome. This miRNA, however, was not upregulated in whole mosquitoes. Also in *Ae. albopictus*, the abundance of 41 miRNAs were significantly altered following chikungunya virus (CHIKV) infection using small RNA deep sequencing [42]. Accordingly, the majority of these miRNAs were downregulated. However, among those, four miRNAs miR-100 (0.3-fold), miR-283 (0.4-fold), miR-305-3p (1.5-fold) and miR-927 (1.9-fold) were identified as upregulated and four miRNAs miR-1000 (2.2-fold), miR-2b (1.4-fold), miR-2c-3p (2.4-fold) and miR-190-5p (1.5-fold) were repressed (based on log fold change and *p*-value in deep sequencing libraries) following CHIKV infection. Although the fold changes of none of the abovementioned miRNAs were validated by RT-qPCR and in some instances the changes appear to be very small. Based on a constructed miRNA:mRNA interaction network analysis, protein tyrosine phosphatase SHP2, extracellular signal regulated kinase (ERK1/2) and ubiquitin fusion degradation protein were found as common targets of the upregulated miRNAs and ribosomal protein S9 as the common target of the downregulated miRNAs. These targets were explored in *Ae. aegypti* in the absence of a *Ae. albopictus* genome and were only based on bioinformatics analysis. Therefore, caution should be exercised in the interpretation of the miRNA-target predictions. In this study, it was also found that aae-miR-2940, a mosquito-specific miRNA, was downregulated upon CHIKV infection. However, the mechanism by which this miRNA is suppressed and its significance in the interaction was not explored. More recently, it was shown that aae-miR-2940-5p’s abundance was also selectively reduced in WNV-infected *Ae. albopictus* C6/36 cells [43]. Aae-miR-2940-5p was previously shown to positively regulate the metalloprotease m41 ftsh (MetP) in *Ae. aegypti* mosquitoes (also see Section 6) [31]. Interestingly, it was found that MetP has a positive effect on WNV replication [43]. Depletion of aae-miR-2940 or silencing of MetP both led to reduced levels of WNV titers suggesting that the miRNA facilitates WNV replication by inducing its target gene, MetP. Therefore, it was concluded that downregulation of aae-miR-2940-5p in mosquito cells is part of the host anti-viral response limiting replication of the virus [43]. Consistent with this result, it was previously found that in *Wolbachia*-infected mosquitoes, in which aae-miR-2940 is highly induced (see below), replication of WNV genomic RNA was enhanced, although assembly or secretion of the virus was still hampered in the presence of *Wolbachia* resulting in lower WNV virions released from the infected cells [44].

In a number of alphaviruses transmitted by mosquitoes, knockdown of *Ago1* gene resulted in no change in virus replication suggesting that the miRNA pathway may not have any effect on virus replication. For example, silencing of *Ago1* gene in *An. gambiae* mosquitoes did not change O'nyong-nyong virus replication [45]. Similarly, depletion of *Ago1* in *Ae. aegypti* Aag2 cells had no effect on Semliki Forest virus [46] or CHIKV replication [47]. However, silencing of *Ago2* or *Ago3*, known to be involved in the RNAi and piRNA pathways, respectively, had significant impacts on virus replication. Given that recent evidence suggests that miRNAs could also be loaded into Ago2 [7,8,9,10], if not other Ago proteins, it is likely that in the absence of Ago1, miRNAs are loaded into Ago2. In humans, in which four Ago proteins have been identified, miRNAs are also randomly sorted to individual Ago proteins [48]. It will be interesting to find out if silencing of *Drosha* or *Dicer1* gene, which are not yet known to be involved in the RNAi or piRNA pathways, has any effect on alphavirus replication.

Viruses can also utilize host miRNAs for their own benefit. For example, in American eastern equine encephalitis virus transmitted by mosquitoes, binding of the mammalian host miR-142-3p to the 3’ UTR of the virus genome is important for tissue tropism and suppression of the host innate immune system [49]. The miRNA limits replication of the virus in myeloid cells by interacting with binding sites in the virus 3’UTR. When the binding sites were removed from the virus genome, its replication increased by about 1000-fold. As a consequence of this virus repression, with the resulting induction of innate immunity, neurologic symptoms of the disease are enhanced. Notably, the binding sites for miR-142-3p in the virus genome are also required for efficient replication and infection of the mosquito vector *Aedes taeniorhynchus*. However, it is not known how these binding site sequences facilitate virus infection in mosquitoes. Given that miR-142-3p is not produced in insects, it is possible that a mosquito-specific miRNA may interact with the sequences resulting in strong selection for the sequences; although there could be other explanations such as the presence of RNA secondary/tertiary structures. Of relevance, a recent study that analyzed the secondary structures in the 3’UTR region of a number of flaviviruses showed the presence of duplicated RNA structures and numerous repeated sequences in the region that could impede the insect immune response by serving as molecular sponges to sequester host proteins or miRNAs [50]. Therefore, interaction of cellular miRNAs with viral sequences may affect host specificity, tissue tropism and consequently virus replication in a positive or negative way. 

So far, we only have a very small number of examples of the impact of arbovirus infections on the miRNA of arthropod vectors and even these mainly provide preliminary information in regards to differential expression of miRNAs which await experimental validations to demonstrate their role in arbovirus-vector interactions. Furthermore, it remains to be determined if the differentially abundant miRNAs following infection are the result of host exploitation by the virus or part of the host response to infection. Another area of interest is to find out how common or different are miRNA changes in a particular vector to different viruses that it transmits and in turn whether the same virus elicits similar changes in different vectors. From the little we know, it appears that miR-2b and miR-1000 are both repressed in *Aedes* mosquitoes when infected with DENV-2 and CHIKV. They may be part of a common response to viral infection or alternatively both manipulated by the viruses. Future research is needed to elucidate the role cellular miRNAs play in arbovirus-vector interactions.

### 4.2. Arbovirus-Encoded miRNAs

The skepticism in regards to the production of miRNAs by RNA viruses, as detailed above, has meant that investigations in this area have been very limited. However, since several studies have shown that the production of mature miRNAs (endogenous or exogenous) from RNA viruses is possible, interest in looking for miRNAs or miRNA-like viral small RNAs from RNA viruses has increased. Arboviruses being mainly RNA viruses fall into this group. So far, there have only been two reports demonstrating production of functional miRNA-like small RNAs from arboviruses, WNV and DENV (Table 1), although there have been reports of RNA virus encoded miRNAs from non-arboviruses produced by non-canonical (e.g., [20,51]) as well as canonical pathways [52]. Meanwhile, results from deep sequencing showing the occurrence of viral small RNAs in low copy numbers has led some to believe that WNV and DENV may not produce any miRNAs [53].

Both WNV and DENV belong to the *Flaviviridae* family and are exclusively transmitted by mosquitoes. Their genomes consist of a single positive strand RNA of around 11 kb with one long open reading frame encoding three structural proteins involved in the formation of virions and seven non-structural (NS) proteins mainly involved in virus replication but also in virus assembly and suppression of the host antiviral response (reviewed in [54,55]). They have a type I cap at the 5’ end but lack a 3’ poly-A tail. The 5’ and 3’ untranslated regions (UTR) of flaviviruses, including WNV and DENV, contain a number of highly structured regions [56], for some of which functions have been assigned (reviewed in [54,57]). From the 3’UTR, a highly structured subgenomic flavivirus RNA (sfRNA; 525 nts in WNV) is produced by almost all members of this family which has essential functions in replication and viral pathogenicity [58,59]. In addition, sfRNA from WNV and DENV were shown to suppress the host RNAi response and interfere with the miRNA silencing pathway in both mammalian and insect cells [60]; although the mechanism of suppression remains to be shown. A recent analysis showed the presence of duplicated RNA structures in the 3’UTR of a number of flaviviruses that are represented as loops/bulges. These were proposed to benefit the virus possibly by facilitating dimerization of proteins involved in virus assembly by providing scaffolds or may interfere with the host immune responses by sequestering host proteins or miRNAs by functioning as molecular sponges [50]. Furthermore, given the number of secondary structures in the 5’ and 3’ regions of flavivirus genomes, the possibility of stem-loop structures serving as precursors for the production of functional small RNAs exists.

Bioinformatics analysis of the WNV sfRNA followed by Northern blot detection and molecular cloning led to the identification of a 21 nt miRNA, KUN-miR-1, from the terminal stem-loop structure (3’SL) in the Kunjin strain of WNV [61]. KUN-miR-1 was expressed in Dicer-2 deficient C6/36 cells, Dicer-2 silenced cells but not in Dicer-1 silenced Aag2 cells confirming that it is not an RNA degradation product. The mature miRNA was also produced from pre-KUN-miR-1 cloned in an insect plasmid expression system or Semliki Forest virus (SFV) replicon constructs. Inhibition of the miRNA using synthetic inhibitor of the miRNA (reverse complementary small RNA with 2’O-methylation modification) or deletion of the sequence from the virus genome led to significant reductions in virus replication. On the other hand, transfection of KUN-miR-1 mimic into cells enhanced replication of the WNV mutant incapable of producing the miRNA. These suggested the importance of KUN-miR-1 in replication of the virus. In regards to its effect on the host, it was found that KUN-miR-1 positively regulates the transcript levels of a transcription factor, GATA4, which plays various roles in insect biology, including transactivation of vitellogenin [62,63] and lipophorin receptor fat body genes [64]. Interestingly, silencing of GATA4 using RNAi in mosquito cells led to significant reductions in WNV replication [61] proposing that the induction of the transcription factor via KUN-miR-1 is beneficial to the virus and perhaps an evolutionary adaptation to manipulate the host. It is not known how induction of GATA4 enhances virus replication, but one possibility is that GATA4 may enhance lipid recruitment to the sites of virus replication through the upregulation of lipophorin receptors facilitating formation of virus-induced membranes, which play important roles in flavivirus RNA replication and virus assembly [65,66]. Alternatively, GATA4 may induce transcription of genes that could benefit replication of the virus. Thus, the exact mechanism of GATA4 enhancement of virus replication remains to be investigated. 

Using bioinformatics and deep sequencing analysis, six miRNA-like viral small RNAs (vsRNAs) were identified in DENV-2 infected mosquitoes that mapped to the stem-loops in the 5’UTR (one vsRNA) and 3’UTR (five vsRNAs) of the virus genome [67]. Transfection of the synthetic miRNA inhibitors of the vsRNAs into Aag2 and RML-12 (from *Ae. albopictus*) cells prior to virus exposure led to significant increases in DENV-2 replication only in the case of vsRNA-5. This 23-nucleotide vsRNA mapped to the very first stem-loop in the 3’UTR. These small RNAs, including vsRNA-5, appeared in low copy numbers in deep sequencing; hence, vsRNA-5’s significance was questioned suggesting that it is an RNAi degradation product [68]. However, several lines of evidence suggest that it may be functional [69]. For example, the precursor and mature vsRNA-5 were detectable in Northern blots (commonly used for validation of miRNAs) from five days following infection in mosquitoes, Aag2 and C6/36 (Dicer-2 deficient) cells [67]. Further, silencing of Dicer-2 did not reduce vsRNA-5 levels, however, silencing of Dicer-1, Ago1 and in particular Ago2 significantly reduced the small RNA levels indicating the involvement of miRNA biogenesis major proteins in its production and stability. The mature vsRNA-5 was also produced from the* in vitro* synthesized single stranded precursor stem-loop RNA transfected into mosquito cells, or when the precursor was overexpressed from a plasmid in the cells independent of the virus. In contrast to the effect of the vsRNA inhibitor in improving virus replication, application of the vsRNA-5 mimic led to reductions in DENV-2 replication. 

Bioinformatics analysis followed by experimental validation showed the presence of target sequences of vsRNA-5 in the non-structural protein 1 (NS1) region. Considering that one long transcript is produced during DENV replication, which consists of structural as well as non-structural proteins, targeting NS1 by vsRNA-5 basically means that the virus genome is targeted. A follow up bioinformatics analysis revealed that vsRNA-5 and its NS1 target sequences might be mainly conserved in DENV serotype 2 and not in the other serotypes [70].

In all instances found so far in which virus genes are the targets of virus-encoded miRNAs, the miRNA-virus interaction leads to regulation of virus replication. For example, miR-BART2 from Epstein Barr virus [71], HvAV-miR-1 from *Heliothis virescens* ascovirus [72], and miR**–**H2-30 and miR-H6 from *Heliothis zea* nudivirus 1 [73] all target viral genes regulating replication of the virus. Given the importance of maintaining the health and fitness of the arthropod vector, it would be logical that an arbovirus auto-regulates its own replication at some stage during its replication to avoid over-replication and quick demise of the vector. This is especially important for biologically transmitted viruses that replicate in a number of tissues before they reach the salivary glands where they further replicate prior to delivery to the next vertebrate host.

**Table 1:** miRNAs with validated function in arbovirus-host interactions.

Origin	Name	Function	Insect species/cell type	References
**Host miRNAs**				
	miR-375	regulates REL1 and cactus genes following blood meal enhancing DENV-2 replication	*Ae. aegypti*	[28]
	miR-2940	regulates metalloprotease ftsh and Dnmt2 genes; enhances DENV-2 and WNV replication	*Ae. aegypti* Aag2 and C6/36 cells	[31,43,74]
**Viral miRNAs**				
	KUN-miR-1	upregulates cellular GATA4 and facilitates WNV replication	Aag2 and C6/36 cells	[61]
	DENV-vsRNA-5	regulates DENV replication by targeting NS1 sequences	Aag2/RML12	[67]

## 5. Blood Feeding and Its Effects on Arboviruses

Taking a blood meal by an arthropod vector results in massive changes in the expression of a large number of genes, including miRNAs, that are involved in reproduction and blood-meal processes (e.g., [38,75,76,77,78,79]). At the same time, arboviruses gain entrance into the vector’s alimentary canal through blood meals. Therefore, it is apparent that selection might favor the pleiotropic effects of pathways involved in blood digestion as well as anti-viral responses. An example is the extracellular-signal-regulated kinase (ERK) signaling pathway, which is induced after a blood meal and is involved in anti-viral responses [80]. Inhibition of the pathway results in an increase in SINV and vesicular stomatitis virus replication in *Drosophila melanogaster* flies and Aag2 cells. 

As demonstrated in *Ae. aegypti*, it appears that there are differences among strains of mosquitoes in regard to their gene expression profiles after blood feeding, thought to be a result of differential gene regulation [81]. Overall, among many other transcript changes, immunity related transcripts, in particular anti-microbial peptides, decreased and those associated with antioxidant activity increased after blood uptake [81]. These could potentially be due to increases in the transcription of inhibitors of the pathways. For example, cactus, an inhibitor of the Toll pathway was found to increase by 4.5-fold (3 days after blood meal) and REL1, an activator of the pathway was downregulated by 5.5-fold after blood meal in *Ae. aegypti* [28]. Similarly, caspar, a negative regulator of the IMD pathway, was found in higher abundance (4.72-fold) after blood uptake in *Ae. aegypti* [78]. These would potentially benefit replication of arboviruses in the absence/decreased levels of immune effector molecules. For example, silencing of the *cactus* gene in *Ae. aegypti* led to 4-fold improvement in replication of DENV in the mosquito midgut [82]. The downstream anti-microbial peptides induced through the Toll pathway may also negatively impact virus replication as was shown in the case of cecropin produced in the salivary glands of *Ae. aegypti* limiting DENV replication [83]. In fact, *cactus* and *REL1* genes were found to be the targets of the blood-induced aae-miR-375 [28] (Table 1). Injection of the synthetic mimic of the miRNA into mosquitoes led to 6-fold increased transcript levels of *cactus* (positive regulation) and 3-fold reduced *REL1* levels (negative regulation) as compared with the control mimic-injected mosquitoes. Interestingly, DENV-2 replicated more efficiently in the presence of aae-miR-375 mimic as compared to controls, which suggested that alterations in miRNA levels following blood feeding might have an indirect impact on replication of arboviruses.

DENV has also been shown to suppress immune related genes including immune signaling proteins and antimicrobial peptides such as cecropin and defensin in Aag2 cells and *Ae. aegypti* mosquitoes when analyzed at the transcriptional level [84]. Other arboviruses, such as SFV and SINV, have also been demonstrated to interfere with mosquito immune responses by inhibiting key immune signaling pathways such as the Toll pathway [85,86]. However, the mechanism of this immune inhibition is not known. Considering the extent of transcriptional changes upon blood feeding, further research is required to investigate the role of differentially expressed genes and miRNAs in arbovirus-vector interactions.

## 6.* Wolbachia*-Mediated Alterations of miRNAs and Their Effects on Arbovirus Replication

Considering the lack of available vaccines and drugs against most arboviruses, management of the diseases caused by arboviruses has heavily concentrated on controlling vector populations or inhibiting virus-transmission by the vectors. Issues with chemical insecticides such as the development of resistance in vector populations, affecting non-target insects and environmental contamination have made non-chemical control alternatives more attractive. These include application of microbial control agents such as entomopathogenic bacteria and fungi, or utilization of symbionts. *Wolbachia* is a prevalent endosymbiotic bacteria found in over 40% of arthropod species [87,88]. Due to the cytoplasmic incompatibility effect (infected males mating with uninfected females produce no viable offspring) imposed by *Wolbachia*, infected females have an advantage, and the endosymbiont rapidly spreads and establishes in the population [89]. In two breakthrough studies, it was shown that *Wolbachia* inhibits replication of RNA viruses *in D. melanogaster* [90,91]. This led to the examination of *Ae. aegypti* mosquitoes transinfected with the *w*MelPop strain of *Wolbachia* in which the life-shortening effect of *Wolbachia* was being explored [92]. Notably, in *Wolbachia*-infected mosquitoes replication of DENV and CHIKV as well as filarial nematode and *Plasmodium* was inhibited [93]. Due to the pathogen blocking property of “some strains” of *Wolbachia*, several attempts have been made to transinfect *Wolbachia* into mosquitoes to produce pathogen refractory mosquitoes (reviewed in [94]). Although no single mechanism has been identified that is responsible for blocking virus replication in *Wolbachia*-infected insects, several factors, including miRNA manipulation, have been identified (see below) that contribute towards this anti-viral property; reviewed recently by Rainey *et al*. [95]. It is worth mentioning that the effect of *Wolbachia* on virus replication may vary from inhibition [90,91,93], no effect [96] to even enhancement [97] depending on the *Wolbachia* strain, the host (natural or transinfected), and the virus. Strikingly, a recent study showed that the *w*AlbB *Wolbachia* strain enhanced WNV replication in transiently transinfected *Culex tarsalis* mosquitoes [97]. This is consistent with an observation made in *Ae. aegypti* Aag2 cells infected with *w*MelPop strain of *Wolbachia*, in which replication of WNV genomic RNA was enhanced as compared to non-infected cells, however, virus secretion and/or assembly in this host (cell line and whole mosquitoes) was inhibited leading to less virion secretion [44]. 

**Figure 2:**
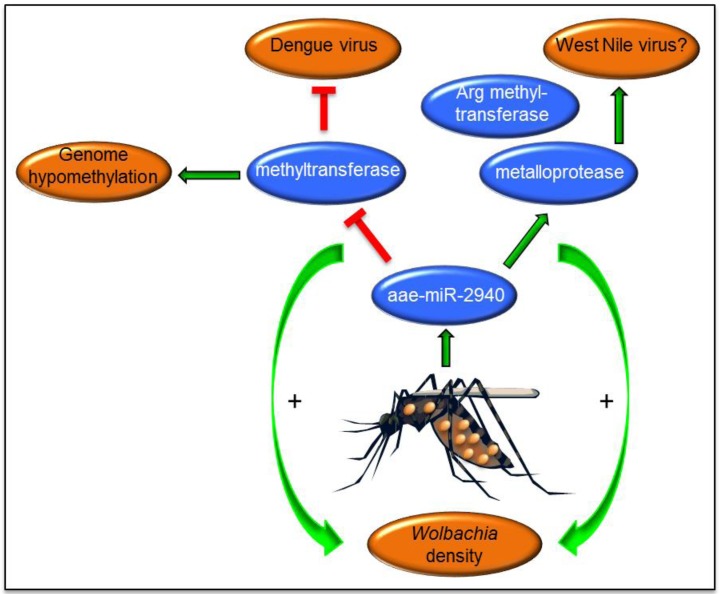
aae-miR-2940-5p is a mosquito-specific miRNA that is highly induced in *Wolbachia*-infected mosquitoes. Three targets of this miRNA validated so far are metalloprotease ftsh, DNA methyltransferase Dnmt2 and Arginine methyltransferase 3 that are important in *Wolbachia* maintenance and/or affect arbovirus replication. Green arrows indicate induction/enhancement, whereas red colour indicates inhibition.

Microarray analysis of miRNAs from female *Ae. aegypti* mosquitoes with and without *w*MelPop *Wolbachia* infection revealed that about 13 miRNAs were differentially expressed in *Wolbachia*-infected mosquitoes. The mosquito-specific miRNA, aae-miR-2940, was highly induced and exclusively detected (using Northern blot analysis) in *Wolbachia*-infected mosquitoes. Initially, MetP was determined as one of the targets of the miRNA and was shown to be important for replication/maintenance of *Wolbachia* [31] (Table 1). The target gene was positively regulated by the miRNA. Inhibition of aae-miR-2940 with miRNA inhibitors or silencing of the MetP gene both led to reductions in *Wolbachia* density. Interestingly, in a subsequent study it was found that MetP enhances WNV replication in Aag2 cells [44]. This perhaps explains the enhanced replication of WNV in *Wolbachia*-infected mosquitoes and mosquito cell lines [44,97] since *Wolbachia* induces MetP expression via induction of aae-miR-2940 (Figure 2); although this needs to be confirmed in *C. tarsalis*. Notably, no significant immune induction was detected in *w*AlbB transinfected *C. tarsalis* mosquitoes [97].

Subsequently, the second target of aae-miR-2940 was identified as the mosquito’s DNA methyltransferase Dnmt2 gene that is negatively regulated by the miRNA [74]. Interestingly, it was found that in mosquito cells without *Wolbachia* infection, Dnmt2 is substantially induced following DENV-2 infection. Overexpression of Dnmt2 by its ectopic expression from a plasmid vector led to increases in DENV-2 replication suggesting that the gene is beneficial for DENV replication. However, since *Wolbachia* suppresses Dnmt2 levels, this contributed to reduced replication of DENV-2. Overexpression of Dnmt2 led to reductions in *Wolbachia* density indicating that the protein has a negative impact on *Wolbachia* and therefore it is suppressed. Alteration of Dnmt2 via aae-miR-2940 appears to affect genome methylation of the host mosquito as experimental evidence confirmed differential methylation, and hypomethylation in general, in the *Ae. aegypti* genome [98], which may have a profound impact on the repertoire of genes transcribed in *Wolbachia*-infected insects. In addition, arginine methyltransferase was determined as another target of aae-miR-2940 [99]. The target gene is positively regulated by the miRNA and its upregulation appears to benefit *Wolbachia* replication, but not DENV. These indicate the significance of aae-miR-2940 in the maintenance of *Wolbachia* and the anti-viral response against DENV (Figure 2). In addition, as noted above, down-regulation of aae-miR-2940 in mosquito cells may potentially serve as an anti-viral response limiting replication of WNV [43]. *Wolbachia* also interferes with the distribution of miRNAs within the host cells [8,100], which may have an effect on regulation of gene expression and consequently impact on virus replication. There are still a number of other miRNAs that are significantly regulated in the presence of *Wolbachia* that have not been explored yet. Further studies should reveal their possible role in *Wolbachia*-vector-virus interactions.

## 7. Evolution of miRNAs in Mosquito Vectors

Based on evolutionary studies carried out in *D. melanogaster*, miRNAs have a high turnover in that there is a high birth rate of new miRNAs as well as a high death rate [101]. New miRNAs emerge through duplication of conserved miRNAs followed by subsequent divergence, or from a non-miRNA sequence and evolve adaptively [102]. Although comparative analysis has not been carried out in any of the arbovirus vectors, a comprehensive report showed that unlike *Drosophila*, in *Ae. aegypti* the major genes involved in both siRNA and miRNA pathways (*Dicer-1*, *Dicer-2*, *Ago1*, *Ago2*, *r2d2* and *r3d1*) undergo rapid, positive and diversifying selection, and that refractoriness of mosquitoes to DENV infection positively correlates with nucleotide diversity indices in the *Dicer-2* gene [103]. This may relate to the biology of mosquitoes as vectors of arboviruses and consequently their continuous infections and exposures to different types of biologically transmitted pathogens and viruses that may serve as sources of diversifying selection detected in mosquito populations.

## 8. Conclusions

Arboviruses of medical and veterinary importance continue to impose large numbers of deaths, health risks and financial losses around the world. In many instances, there are no effective drugs or vaccines available for diseases caused by arboviruses. Despite many groups around the world working on arboviruses, there are still many gaps in our knowledge in regards to the interaction of arboviruses with their hosts, in particular, the arthropod vector. Deeper understanding of molecular mechanisms regulating interplaying events such as blood-feeding, reproduction and anti-viral responses may lead to development of new approaches in arbovirus control through blocking/limiting transmission of arboviruses by arthropod vectors. 

MiRNAs have appeared as key molecules in the regulation of gene expression at both transcriptional and post-transcriptional levels, playing important roles in development and response of animals to environmental stimuli. Current knowledge, although limited, points to the fact that miRNAs are involved in the regulation of various aspects of arbovirus-vector interactions. However, we have only scratched the surface. Many differentially expressed miRNAs upon blood feeding, exposure to arbovirus infections, and at different developmental stages, have been identified, but their significance in those events or interactions have not been experimentally tested. Considering the short length of miRNAs and the absence of host immune responses to miRNA, they are ideal for incorporation into innovative strategies to control arboviruses; for example, generating genetically modified vectors that are refractory to virus infection by overexpressing miRNAs that boost the host anti-viral response or directly target arboviruses. 

The discovery of the virus blocking property of *Wolbachia* in arthropod vectors has provided a unique opportunity to explore the endosymbiont in control of arboviruses and in fact other arthropod-borne pathogens [94,104]; although caution must be exercised since the endosymbiont may have varying effects on virus replication, including enhancement [97]. Exploration of the role of miRNAs, as master regulators of gene expression, in conferring protection to insects against arboviruses may pave the way to better manage the interaction and optimize its anti-viral property or avoid its loss from newly introduced vector populations.
